# Induction of Encephalitis in Rhesus Monkeys Infused with Lymphocryptovirus-Infected B-Cells Presenting MOG_34–56_ Peptide

**DOI:** 10.1371/journal.pone.0071549

**Published:** 2013-08-15

**Authors:** Krista G. Haanstra, Jacqueline A. M. Wubben, Margreet Jonker, Bert A. ‘t. Hart

**Affiliations:** 1 Department of Immunobiology, Biomedical Primate Research Centre, Rijswijk, The Netherlands; 2 Department of Neuroscience, University Medical Center, Groningen, The Netherlands; Washington University, United States of America

## Abstract

The overlapping epidemiology of multiple sclerosis (MS) and Epstein-Barr virus (EBV), the increased risk to develop MS after infectious mononucleosis (IM) and the localization of EBV-infected B-cells within the MS brain suggest a causal link between EBV and MS. However, the underlying mechanism is unknown. We hypothesize that EBV-infected B-cells are capable of eliciting a central nervous system (CNS) targeting autoimmune reaction. To test this hypothesis we have developed a novel experimental model in rhesus monkeys of IM-like disease induced by infusing autologous B-lymphoblastoid cells (B-LCL). Herpesvirus papio (HVP) is a lymphocryptovirus related to EBV and was used to generate rhesus monkey B-LCL. Three groups of five animals were included; each group received three intravenous infusions of B-LCL that were either pulsed with the encephalitogenic self peptide MOG_34–56_ (group A), a mimicry peptide (981–1003) of the major capsid protein of cytomegalovirus (CMVmcp_981–1003_; group B) or the citrullinated MOG_34–56_ (cMOG_34–56_; group C). Groups A and B received on day 98 a single immunization with MOG_34–56_ in incomplete Freund’s adjuvant (IFA). Group C monkeys were euthanized just prior to day 98 without booster immunization. We observed self-peptide-specific proliferation of T-cells, superimposed on similar strong proliferation of CD3^+^CD8^+^ T-cells against the B-LCL as observed in IM. The brains of several monkeys contained perivascular inflammatory lesions of variable size, comprising CD3^+^ and CD68^+^ cells. Moreover, clusters of CD3^+^ and CD20^+^ cells were detected in the meninges. The only evident clinical sign was substantial loss of bodyweight (>15%), a symptom observed both in early autoimmune encephalitis and IM. In conclusion, this model suggests that EBV-induced B-LCL can elicit a CNS targeting inflammatory (auto)immune reaction.

## Introduction

Multiple sclerosis (MS) is a progressive neuro-inflammatory disease affecting the central nervous system (CNS) of about 1 per 1000 young adults in Western societies. The pathological hallmark of MS and the most likely cause of the neurological deficit is the lesion, being a usually focal area of demyelination in white and grey matter characterized by a variable degree of inflammation, injury to neuro/axonal complexes and proliferation of astrocytes (gliosis) [1], [2]. According to a widely accepted concept, lesions in the CNS of MS patients are formed by the synergy of humoral and cellular autoimmune reactions against CNS components. These are thought to develop in genetically susceptible individuals in response to infection with as yet unidentified pathogens [3], [4]. The similar epidemiology of MS and Epstein-Barr virus (EBV) infection, as well as the increased risk to MS after infectious mononucleosis (IM), the symptomatic form of EBV infection, point to EBV as an important environmental trigger of MS (reviewed in [5]). MS patients are more frequently infected with EBV than people without MS, i.e. 99% versus 94% in adults [6] and 83–99% versus 42–72% in children [7], [8]. Moreover, the risk to develop MS is about 2 to 3-fold increased in EBV positive individuals with a history of infectious mononucleosis (IM) as compared to EBV positive individuals without a history of IM [6].

It is not possible to directly investigate the causal relation between IM and MS in humans, since over 90% of the population is already infected with EBV and for obvious ethical reasons, virus negative humans cannot be infected with the virus. EBV is a member of the γ-herpesvirus family of lymphocryptoviruses (LCV). LCV are rather species specific, but conserved parts of the genome can be found in LCV infecting other mammal species. However, the biological functions are remarkably similar between species [9]–[11]. Studies on the effects of LCV in its target species can serve as a representative model to study the effect of EBV infections in humans [12]–[14]. The effect of latent LCV infection on the development of experimental autoimmune encephalitis (EAE), the experimental model for MS, was elegantly studied in the mouse, using a mouse relative of EBV, γHV-68 [15]. Infected mice developed accelerated and more severe EAE, with a Th1 skewing of the response. Remarkably, while inflammation and demyelination in non-infected EAE mice was predominantly found in spinal cord, virus-infected EAE mice displayed pathology also in the brain.

The aim of the current study was twofold: 1. To test whether *in vivo* presentation of self-antigen by a non-human primate relative of EBV, *herpes virus papio* (HVP)-transformed autologous B-lymphoblastoid cells (B-LCL) elicits activation of self-antigen specific T cells and 2. To test whether this would lead to neuroinflammation. The experiments were conducted in rhesus monkeys, a non-human primate species closely related to humans which has frequently been used as an autoimmune model of MS [16]. We have targeted our research on the activation of T-cells specific for peptide 34–56 from the CNS antigen myelin oligodendrocyte glycoprotein (MOG_34–56_). This peptide encompasses the most relevant T-cell epitopes for EAE induction in two non-human primate species, i.e. rhesus macaques [17] and common marmosets [18]. T-cells specific for this peptide have the remarkable capacity to induce widespread demyelination without the support of antibodies. Moreover, they display characteristics of anti-viral memory cells [19], which is supported by the clear crossreactivity with a peptide from the immuno dominant major capsid protein (ORF UL86) of cytomegalovirus (CMV) and MOG_34–56_ [17].

For the current study in rhesus monkeys we prepared autologous B-LCL lines by infection of peripheral blood B-cells with HVP, being the most closely related non-human primate LCV to EBV [20]. EBV itself could not be used as the capacity to infect macaque B-cells is very low [21], [22]. Prior to infusion into the autologous monkey, the B-LCL were pulsed *ex vivo* with peptides that activate MOG_34–56_ T-cells, i.e. in group A the encephalitogenic peptide MOG_34–56_, in group B the CMV-derived peptide CMVmcp_981–1003_, which activates T-cells specific for MOG_34–56_ but fails to induce EAE [17] and in group C citrullinated MOG_34–56_ (cMOG_34–56_). Citrullination is a common post-translational modification of proteins associated with inflammation, where arginines are converted into citrulline by deimination. Peptidylarginyl deiminase (PAD) enzymes 2 and 4 were upregulated in the brains of mice with EAE [23]. Immunization of mice with citrullinated MOG_35–55_ induced pathogenic T-cell subsets that could contribute to pathogenic process induced by T-cells induced with the native form of MOG_35–55_ [23]. Citrullination of myelin has been detected in MS patients, indicating that the encephalitogenic T-cells may recognize citrullinated self-peptides [24]. We included group C (cMOG_34–56_) to investigate if this would lead to an increase in pathogenic responses.

The results show that PBMC from the B-LCL infused monkeys displayed a variable proliferative response of mainly CD3^+^CD8^+^CD56^+^ and CD3^−^CD56^+^ lymphocytes against the B-LCL. Superimposed on the response against the B-LCL we observed proliferation against the pulsed peptides in several monkeys, provided that these were presented in the *ex vivo* assays by autologous B-LCL. *Post mortem* immunohistological examination of the brain white matter showed in all group C monkeys and several monkeys of groups A and B the presence of small to medium-sized perivenular infiltrates of CD3^+^ T-cells and CD68^+^ macrophages. In the meninges of several monkeys clusters of CD3^+^ and CD20^+^ lymphocytes were found.

## Materials and Methods

### Ethics Statement

Adult rhesus monkeys (*Macaca mulatta*) were purchased from a licensed breeder. All procedures were performed in compliance with guidelines of the Institutional Animal Care and Use Committee (IACUC) of the Biomedical Primate Research Centre (BPRC) in accordance with Dutch law. This study was approved by the IACUC under permit number DEC#577. Animals were housed at the BPRC. BPRC’s policy on the welfare of experimental animals can be found at http://www.bprc.nl/en/animal-welfare/. Some animals were pair housed, most were housed single, but all in one room. Monkey chow and water were provided ad libitum, supplemented with fresh fruit and vegetables, divided over several times per day. Animals were observed daily for signs of discomfort and suffering, but no abnormalities were noted. Animal handling was performed under ketamine sedation. Animals were sacrificed by an overdose of the barbiturate pentobarbital.

### Antigens

Recombinant human MOG 1–125 (rhMOG) was produced in house from E. coli expressing the extracellular domain of human MOG as transgene. The transfected bacteria were purchased from dr. Hans van Noort (TNO Prevention and Health, Leiden, The Netherlands).

Three synthetic peptides were used: human MOG_34–56_ (GMEVGWYRPPFSRVVHLYRNGKD; CRB, Cleveland, UK), CMVmcp_981–1003_ (HEYHNWLRSPFSRYSATCPNVLH [25]; ABC, London, UK). cMOG_34–56_ (GMEVGWYXPPFSXVVHLYXNGKD, where X stands for citrulline) was purchased from J.W. Drijfhout (LUMC, Leiden, The Netherlands).

### Experimental Outline

Fifteen aged monkeys were stratified over 3 groups of 5 monkeys each (Table 1), to ensure balanced age, weight and sex distribution and to divide animals with well-growing B-LCL equally over the groups. Peripheral blood mononuclear cells (PBMC) from each monkey were collected under ketamine anesthesia (10 mg/ml/kg; AST Pharma, Oudewater, The Netherlands) from the femoral vein and infected *in vitro* with baboon LCV (*Herpesvirus papio*) [22]. B-LCL were harvested and counted. The yield was highly variable (Table 1) which was often due to growth inhibition by foamy virus present in the cultures, in spite of the addition of the antiretroviral drug PMPA (Gilead Sciences, Foster City, CA, USA) [22]. In cases where high numbers of B-LCL were obtained, dead cells were removed by density gradient centrifugation (Table 1, bold numbers). In other cases, this enrichment step was skipped. A maximum of 50×10^6^ B-LCL were pulsed overnight at 37 °C with 6.25 µg/ml peptide. B-LCL from animals in group A were pulsed with MOG_34–56_, B-LCL from animals in group with CMVmcp_981–1003_ and B-LCL from animals in group C with cMOG_34–56_ (Table 1). We chose not to use B-LCL without pulsing peptide as control, as in that case peptide-specific cellular immune responses could not be monitored. Instead, the monkeys in the control group B were administered B-LCL pulsed with the cytomegalovirus major capsid protein-derived peptide CMVmcp_981–1003_. The rationale was that immunization of rhesus monkeys with this mimicry peptide of MOG_34–56_ formulated in CFA induced activation of MOG_34–56_ T-cells, but unlike MOG_34–56_ did not induce EAE [17].

**Table 1:** Animal demographics and identification with B-LCL doses.

Group	Animal		Age (years)	Weigh (kg)	Sex	B-LCL dose		
	ID	Code				IV1	IV2	IV3
A (MOG_34–56_)	02	A1	14.7	6.5	F	3.0	8.6	21.0
	N78	A2	14.1	6.0	F	10.2	50.0	**50.0**
	N85	A3	15.0	7.0	F	38.5	**42.0**	**44.4**
	Ri121	A4	13.3	5.65	M	**40.0**	**50.0**	**50.0**
	Ri284	A5	10.8	10.8	M	**50.0**	**50.0**	**50.0**
*Average*			*13.6*	*7.2*		*28.3*	*40.1*	*43.1*
B (CMVmcp_981–1003_)	03	B1	14.4	6.2	F	**50.0**	**50.0**	**26.0**
	N79	B2	14.0	6.5	F	22.2	50.0	**50.0**
	N91	B3	14.5	6.0	F	32.5	4.3	5.5
	Ri059	B4	13.3	5.7	F	12.6	1.2	2.9
	Ri440	B5	11.1	12.5	M	4.2	2.5	17.9
*Average*			*13.5*	*7.4*		*24.3*	*21.6*	*20.5*
C (cMOG_34–56_)	N77	C1	14.5	6.0	F	4.8	2.5	12.4
	N96	C2	14.1	5.7	F	35.9	**50.0**	**50.0**
	N99	C3	14.4	5.7	F	14.0	2.9	1.2
	Ri107	C4	16.2	11.2	M	21.1	38.0	**33.6**
	Ri12212	C5	9.7	4.9	F	**50.0**	49.5	8.5
*Average*			*13.8*	*6.7*		*25.2*	*28.6*	*21.1*
P value			0.77	0.47		0.96	0.34	0.14

Intravenous (IV)1, IV2, IV3: Doses of infused B-LCL (x10^6^ viable cells) pulsed with the indicated peptides (group A: MOG_34–56_, group B: CMVmcp_981–1003_ and group C: citrullinated MOG_34–56_) are given for each infusion. Values in bold indicate that dead B-LCL were removed before pulsing with peptide by density gradient centrifugation. None of the parameters differed significantly between the groups.

Prior to intravenous injection, harvested B-LCL were washed once with PBS. Intravenous injections with peptide-pulsed autologous B-LCL were administered on days 0, 28 and 56. Hematological parameters were determined on a Sysmex XT-2000i (Sysmex, Norderstedt, Germany) every two weeks for the duration of the experiment, starting prior to the first autologous B-cell transfer. Total lymphocyte numbers were normalized against the individual lymphocyte counts on day 0.

At psd 98 clinical signs of EAE were observed in none of the monkeys. Therefore we chose to sacrifice the monkeys from group C to examine whether histological and immunohistochemical signs of EAE were visible in the brains. To test whether the pre-exposure of the monkeys to peptide-pulsed B-LCL would make them more susceptible to EAE, the animals of groups A and B were boosted with 100 µg MOG_34–56_ dissolved in 500 µl PBS, and emulsified in an equal volume of incomplete Freund’s adjuvant (IFA; Difco Laboratories; Detroit MI). It is of note that in unlike the situation in marmosets, immunization of rhesus monkeys with MOG_34–56_ does not induce clinical signs or pathology of EAE. The animals were euthanized between six and eight weeks after the boost (days 141–154 post first injection).

### Immunohistochemistry on CNS Cryosections

We prepared 6 µm thick sections from snap-frozen tissues (liquid N2), which were air dried and fixed for 10 minutes in acetone. Single staining was performed using peroxidase or alkaline phosphatase labelled antibodies using a Vectastain ABC kit (Vector laboratories, PK-4000) or a double staining using Envision G/2 doublestain system (DakoCytomation, K5361). Slides were pre-incubated with appropriate enzyme blocking reagents to diminish aspecific reactions for avidine, biotine, and peroxidase. The tests were carried out according to the instructions of the suppliers of the kits with minor modifications. The following antibodies were used: CD3 (FN18, BPRC, The Netherlands), CD20, CD68 (clones L26 and KP1, Dako), The slides were counterstained with haematoxylin. Staining patterns of the slides were analysed by a blinded observer. At least 4 slides taken from different parts of the brain were analysed and in each case the whole slide was examined for the presence of stained cells. An animal was scored positive when several cuffs containing 4 or more stained cells per slide were found.

### Cellular Immune Responses

#### Proliferation

PBMC were isolated before the first autologous B-LCL injection, every two weeks thereafter and at the time of necropsy. At necropsy mononuclear cells (MNC) were isolated from the spleen as well as lymph nodes from axillary (ALN) and inguinal (ILN) regions. For [^3^H]-thymidine incorporation assays, cells were dispensed in quadruplicate at 1×10^5^ cells/well in flat-bottom microtiter wells with the relevant antigens. Tested antigens were MOG_34–56_, CMVmcp_981–1003_, cMOG_34–56_ and rhMOG all at 0.1 mg/ml. Stimulation by ConA (5 µg/ml) was used as a positive control. Mononuclear cells were also cultured as described above in the presence of 2×10^5^ autologous B-LCL/well. MNC proliferation was assayed by the incorporation of [^3^H]-thymidine (0.5 µCi/well) during the final 18 hrs of a 5-day culture. Cells were harvested for β-scintillation counting (Topcount NXT, Packard, Ramsey, MN, USA). The results obtained from the different culture conditions were expressed as delta counts per minute (Δcpm):
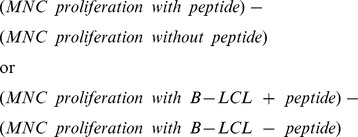



### Phenotype

To determine the phenotype of the proliferating lymphocyte fraction, MNC were incubated for 7 min. at room temperature with carboxyfluoresceindiacetate-succinimidylester (CFSE 2.5 µM; Molecular Probes, Leiden, The Netherlands). This fluorescent vital dye binds cytosolic proteins and the dilution of fluorescence at every cell division can be visualized by flow cytometry. Labelled MNC were cultured with non-labelled B-LCL as described above. After 7-days of culture, a Live/Dead stain (1∶5000, cat# L34955, Invitrogen, Breda, The Netherlands) was performed. Subsequently, cells were stained with antibodies against CD3, CD4, CD8 and CD56 (clones SP34, L200, SK1 and NCAM16.2, respectively, all from BD, San Diego, CA, USA). Fluorescent cells were analyzed using an LSRII equipped with FACSDiva software (BD).

### Humoral Immune Responses

Serum samples were collected prior to immunization, every two weeks thereafter, and at necropsy. The sera were tested by ELISA in 96-well microtiter plates for the presence of antibodies against the three sensitizing peptides MOG_34–56_, CMVmcp_981–1003_, cMOG_34–56_ or against rhMOG. The plates were coated with the antigens (5 µg/ml) and incubated overnight at 4 °C. After washing and blocking with PBS/1% BSA the wells were incubated in duplicate with 1∶100 diluted sera. Binding was detected with alkaline phosphate-labelled goat-anti-human IgG (1∶2000, cat# AHI1305, Invitrogen) or alkaline phosphate-labelled goat-anti-human IgM (1∶10000, cat# A9794, Sigma, Zwijndrecht, The Netherlands). Antibody binding was quantified with p-nitrophenyl phosphate (Sigma). A positive antibody response was defined as the light absorbance at 405 nm being >1.5 times than the absorbance of the day-0 sample on at least two time points.

### Statistics

Statistical analysis was performed using Prism 5 for Mac OS X (GraphPad, San Diego, CA, USA). Data are expressed as mean ± SEM. Significance of differences between groups were calculated with the non-parametric Wilcoxon matched pairs test of the one-way ANOVA (Kruskal-Wallis test). A P≤0.05 was considered significant.

## Results

### Variable Natural Immune Reactivity Against Autologous B-LCL in the Rhesus Monkey Cohort

**Figure 1:**
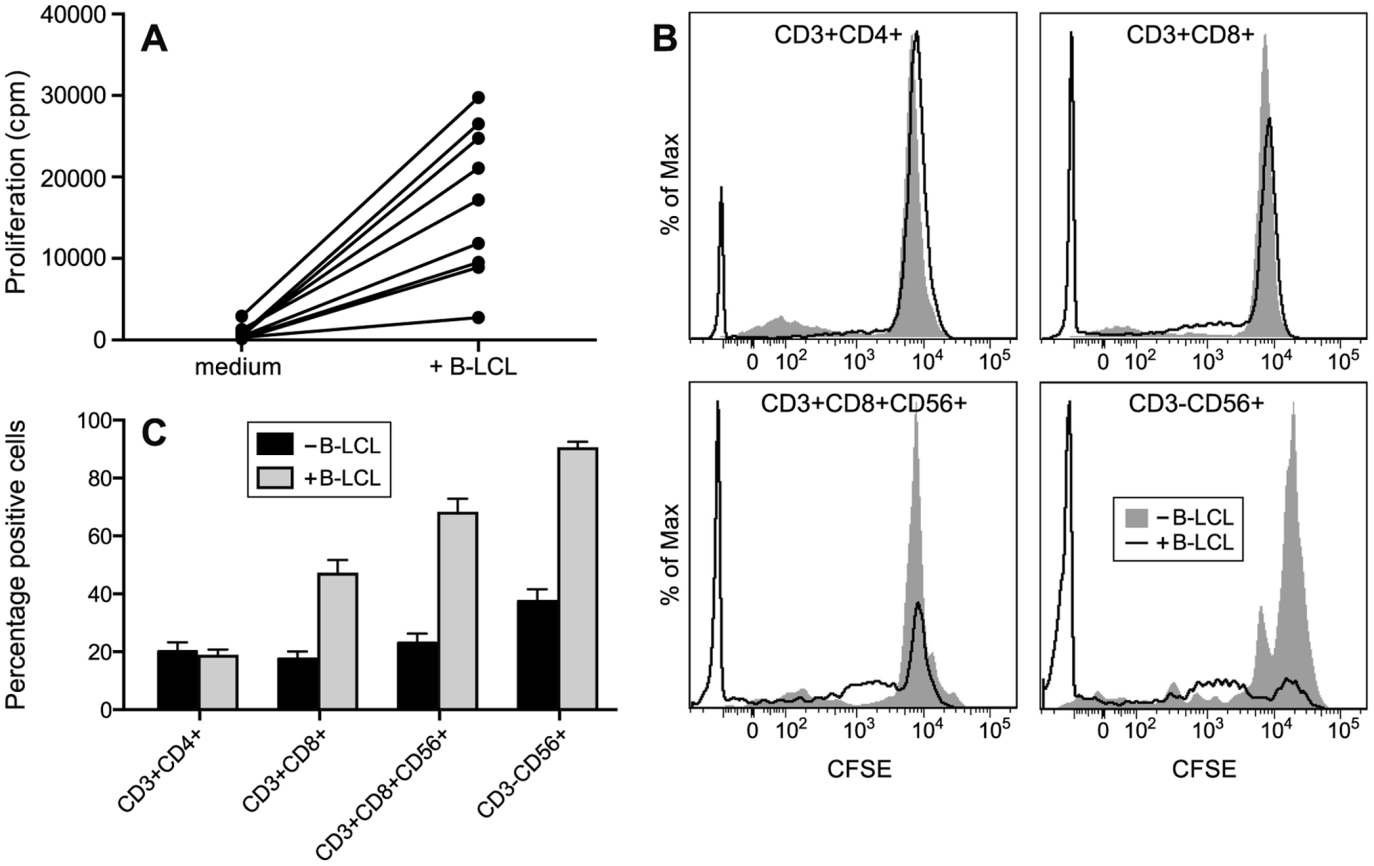
Rhesus monkeys display a naturally occurring cellular immune status against B-LCL. A) PBMC proliferate *ex vivo* when cultured with autologous B-LCL (n = 9). The response increased from 835±288 cpm to 16920±3076 cpm (P = 0.0039; Wilcoxon matched pairs test). B) A representative example (animal A4) of the phenotype of proliferating T-cells (percentage cells that have diluted CFSE) present in the natural repertoire proliferating *ex vivo* against B-LCL. Solid histograms show proliferation in the absence of B-LCL, black lines show proliferation in the presence of B-LCL. C) Mean CFSE dilution in the absence of B-LCL (n = 15; black bars) and in the presence of B-LCL (n = 9). Consistent with the literature on EBV, these are CD3^+^CD8^+^ (regular CTL) and CD3^−^CD56^+^ (NK). Interestingly, also CD3^+^CD8^+^CD56^+^ T-cells proliferate, which are presumably NK-CTL, a subtype that is of interest for the EAE model [18].

Humans have a natural immune status against EBV, mostly consisting of CD8^+^ T-cells that limit dissemination of the virus [26]. The LCV of baboons (*Herpesvirus papio*) that was used for generation of rhesus monkey B-LCL is closely related to the LCV of macaques [21]. Hence, we have screened the selected rhesus monkeys for the presence of a pre-existing T-cell reactivity against the autologous B-LCL. Figure 1A shows that PBMC collected before the B-LCL infusion display a variable proliferative response against B-LCL suggesting that the normal rhesus monkey repertoire contains macaque LCV specific T-cells cross-reacting with antigens from HVP, although the monkeys had never been exposed to HPV. The proliferating fraction was gated with the help of CFSE vital dye dilution and phenotyped with cross-reactive monoclonal antibodies directed against human CD markers. Figure 1B and C demonstrate that the proliferating fraction mainly comprised of CD3^+^CD8^+^ cytotoxic T-lymphocytes, CD3^+^CD8^+^CD56^+^ natural-killer type cytotoxic T-lymphocytes (NK-CTL) and CD3^−^CD56^+^ natural killer cells. Note that only negligible proliferation of CD4^+^ T-cells was observed.

### *Ex vivo* Analysis of the *in vivo* Induced Cellular Immune Reactivity

**Figure 2:**
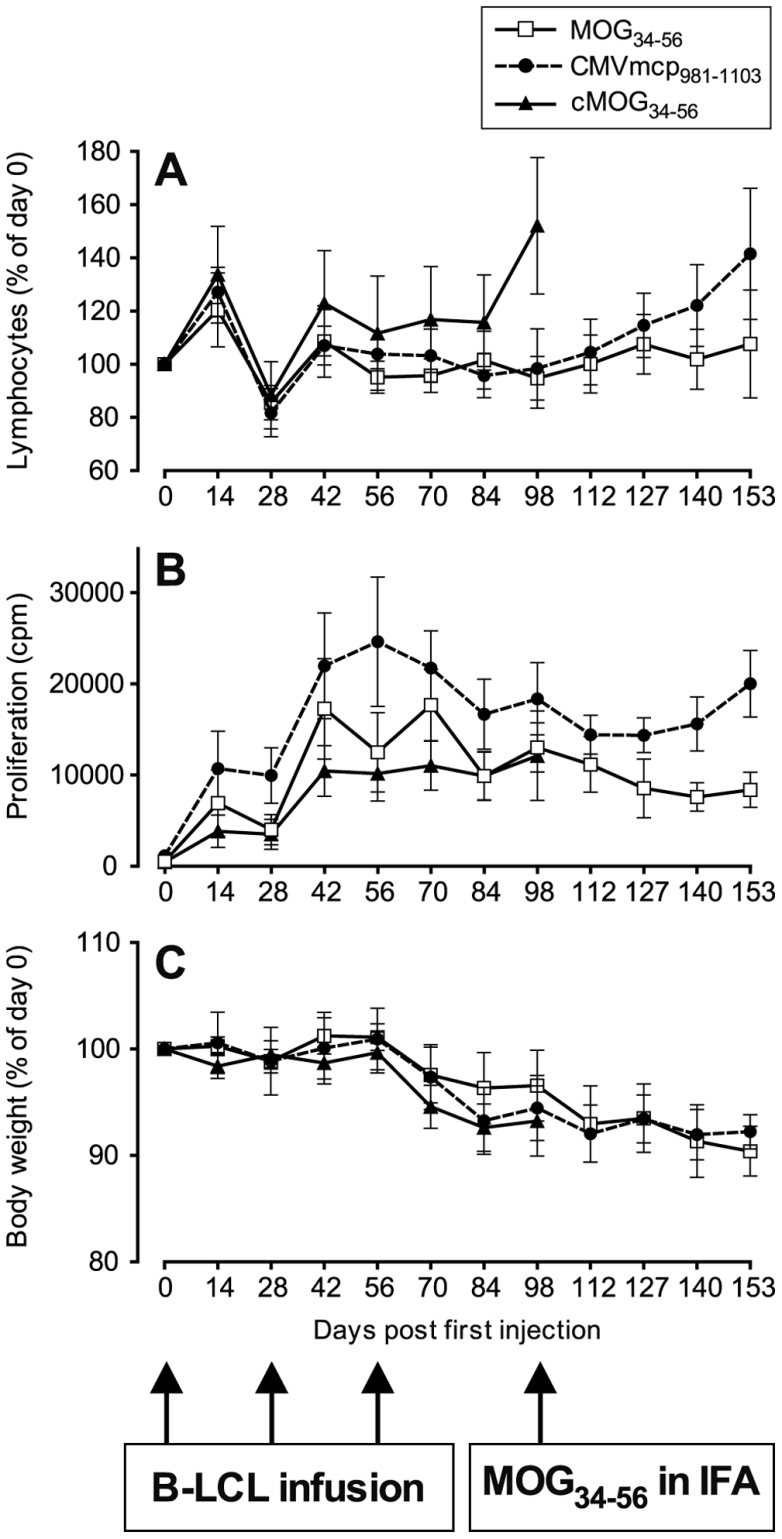
Systemic effects of B-LCL infusion. B-LCL were infused on days 0, 28 and 56. On day 98 animals from groups A (MOG_34–56_) and B (CMVmcp_981–1003_) were immunized with MOG_34–56_ in Incomplete Freund’s adjuvant (IFA). (A) The infusion of autologous B-LCL induces transient increment of circulating lymphocytes. For normalization purposes lymphocyte numbers were expressed relative to day 0 (mean ± SEM 1.8±0.3; 2.4±0.4 and 2.0±0.3×10^9^/l for groups A, B and C respectively, which is not significantly different from each other, P = 0.2184). The increases in numbers of circulating lymphocytes vastly exceeded the number of infused B-LCL. (B) The *ex vivo* proliferation of unstimulated PBMC was persistently increased after the first B-LCL injection. No significant differences were observed between groups (P = 0.2299). (C) The bodyweights were normalized against the day-0 bodyweights. Bodyweight loss is observed from day 70 onwards, up to 15% in some animals.

Autologous B-LCL were infused in variable numbers, depending on the amount of B-LCL that could be harvested from the bulk cultures (Table 1). It has been well established that EBV infection of immunocompetent humans induces a brisk response of CD8^+^ T-cells, resulting in expansion to about 40 to 60% of the CD8^+^ T-cell pool in blood [26], [27]. A similar expansion of lymphocytes was observed in monkeys infused with autologous B-LCL (Fig. 2A). The B-LCL injections seemed to cause a transient expansion of blood lymphocyte numbers with about 27% as compared to pre-infusion blood lymphocyte numbers. This corresponds to an average of 5×10^8^ cells per liter blood. It is unlikely that this increment is only due to the infused B-LCL. The circulating blood volume of a 7 kg rhesus monkey is about 0.5 litres. The infusion of the average of 25 million B-LCL could have induced a maximum increase of 5×10^7^/l lymphocytes, which is about 10-fold less than the observed increment. We conclude therefore that the increment is caused by the vigorous T-cell response against the B-LCL.

To analyse whether the infusion of B-LCL also induced *in vivo* immune activation we determined the *ex vivo* proliferation of PBMC without additional stimuli, which will henceforth be referred to as the “unstimulated” proliferative response. After each infusion with B-LCL a variable increase of the level of “unstimulated” proliferation could be observed (Fig. 2B). The increase of “unstimulated” PBMC proliferation gradually declined with time after the last B-cell infusion, suggesting that the initial expansion of the anti-B-LCL response is followed by contraction. Fig. 2B also shows that whereas B-LCL pulsed with MOG_34–56_ or citrullinated MOG_34–56_ induced a more or less similar “unstimulated” proliferation profile, a higher level of proliferation was observed in monkeys receiving B-LCL pulsed with CMVmcp_981–1003_, although not significantly different from the other two groups. This suggests that the immune system of the recipient monkey may not only detect viral antigens expressed by B-LCL, but also distinguish between the presented peptides used to pulse the B-LCL.

We observed substantial loss of bodyweight exceeding 15% in some animals (Fig. 2C), which is an accepted surrogate marker of clinical EAE and also a symptom of IM. Evident neurological deficits were not observed. We therefore chose to test whether the infusion of MOG_34–56_-pulsed B-LCL may have induced priming of MOG_34–56_-reactive encephalitogenic T-cells in the recipient monkeys without inducing full-blown activation. For this reason the monkeys from groups A and B were given a single booster immunization with MOG_34–56_ in IFA at day 98, which in a previous study was found in itself to be an insufficient stimulus of clinical EAE induction in rhesus monkeys [17]. The booster immunization induced a subacute rebound of the *ex vivo* detected ‘unstimulated’ proliferation of PBMC and an increment of lymphocyte numbers, which was most prominent in the group B monkeys. Note that the monkeys from group C monkeys were not immunized, but were directly sacrificed for neuropathological examination.

**Figure 3:**
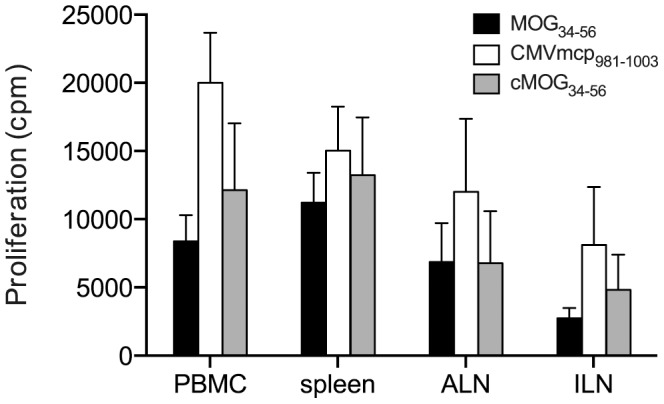
Spontaneous proliferation of unstimulated mononuclear cells (MNC). PBMC and MNC from spleen, axillar (ALN) or inguinal (ILN) lymphnodes were assayed for spontaneous proliferation at the time of necropsy. Most proliferating cells are confined to the peripheral blood and spleen. Similar to the PBMC from animals of group B (CMVmcp_981–1003_; Fig. 2B), splenic and lymph node MNC from group B animals also proliferate more than cells from animals from the other two groups, although this was not significant for any of the cell sources.

At necropsy, the presence of spontaneously proliferating T-cells in blood, spleen as well as the inguinal and axillary lymph nodes was determined in all three groups. Proliferating cells were mostly located in peripheral blood and spleen (Fig. 3). Interestingly, the distribution of proliferating cells in groups A and C was remarkably similar, but the peripheral blood of group B animals contained a considerably higher fraction of the proliferating cells than in the other two groups.

**Figure 4:**
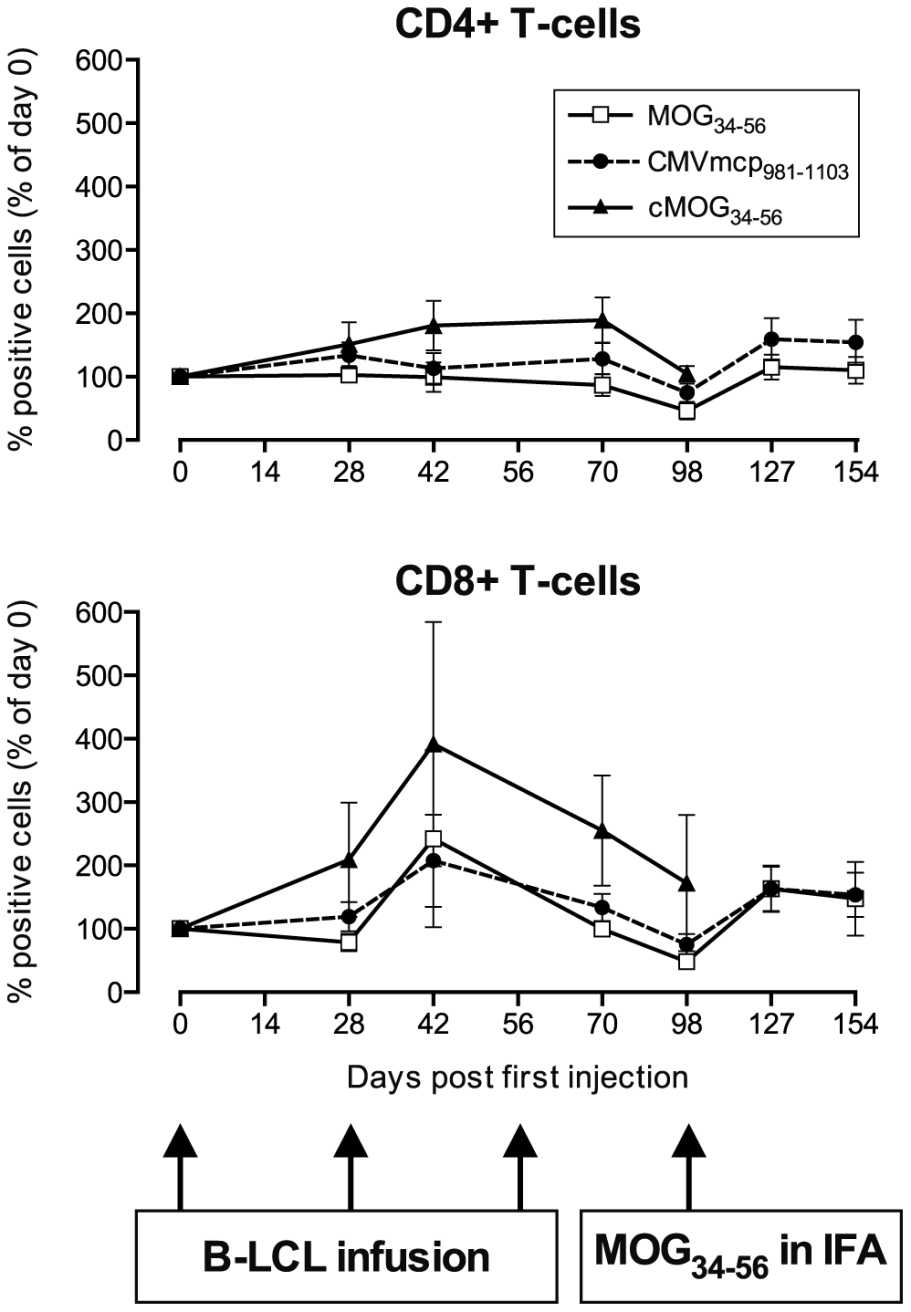
In vivo expansion of T-cells. Percentages of CD4^+^ T-cells and CD8^+^ T-cells in PBMC, expressed as a percentage of day 0, demonstrate that mainly the CD8^+^ subpopulation is expanded after the infusion of B-LCL, which is consistent with the data in Figure 1. Percentages of CD4^+^ T-cells on day 0 were: 35.7±11.2; 36.8±9.8 and 29.5±19% for groups A, B and C respectively, which is not significantly different from each other (P = 0.4677). Percentages of CD8^+^ T-cells on day 0 were: 18.4±3.5; 21.2±5.2 and 10.2±2.5% for groups A, B and C respectively (P = 0.1451). Consistent with our published data in rhesus monkeys [17] an expansion of both CD4^+^ and CD8^+^ T-cells was observed after a booster with MOG_34–56_ in IFA.

The fluctuations of CD4^+^ and CD8^+^ T-cell numbers in PBMC after B-LCL infusion were determined in a longitudinal fashion. In line with the data in Figure 1B, we observed in all three groups a transient elevation of CD8^+^ T-cells in blood, while only in the group C monkeys a simultaneous increase of the CD4^+^ T-cell percentage was observed. Elevation of blood CD4^+^ T-cells in group A and B monkeys was observed only after the immunization with MOG_34–56_ in IFA (Fig. 4).

Taken together, these findings imply that the rhesus monkey T-cells did not only respond to viral peptides presented on the B-LCL, but were also capable to detect the peptides with which the B-LCL had been pulsed.

### Induction of Peptide-specific T-cell Responses

**Figure 5:**
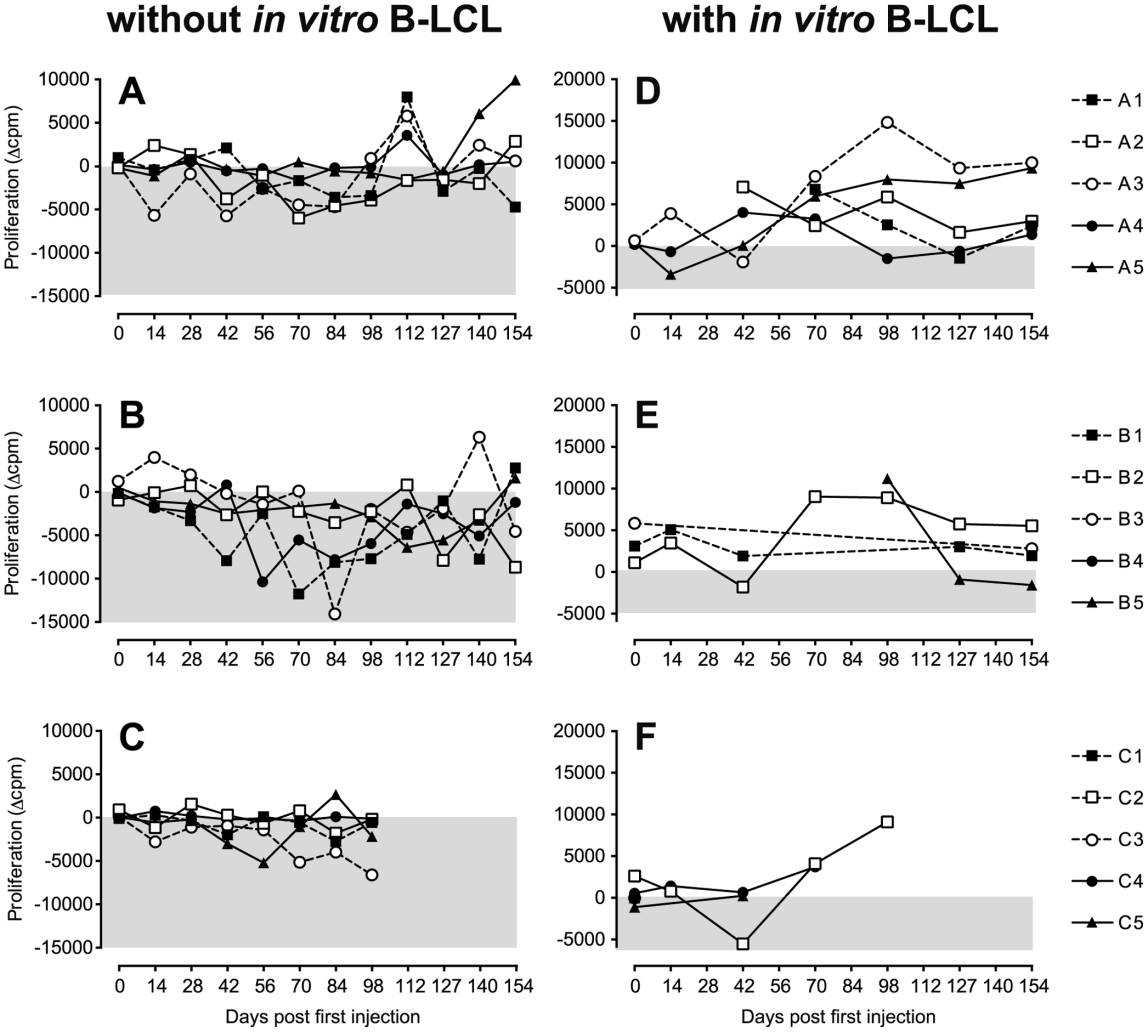
Ex vivo PBMC responses. (A, B, C) peptide-specific PBMC responses, whereby the background response of PBMC without peptide is subtracted (Δcpm). The background response was 710±177 cpm, but increased to 16000 (1000–39000) cpm (mean (range)) around day 42 to 70, where after it decreased slightly (see Figure 2B). (D, E, F) PBMC responses in the presence of peptide pulsed autologous B-LCL, whereby the background response of PBMC+non-pulsed B-LCL is subtracted (Δcpm). The background response was between 2700 and 30000 cpm on day zero (see Figure 1A), but it increased over time. The background response was on average 48000 cpm, and ranged between 10000 and 106000 cpm between days 14 and the end of the experiment. B-LCL were not available in sufficient numbers for all animals for all time points.

The data presented in Figures 2, 3 and 4 show that the three peptides used for pulsing of the B-LCL elicit different patterns of immune reactivity. We therefore investigated whether also peptide-specific T-responses could be detected. These were calculated by subtracting spontaneous proliferation in “unstimulated” cultures (Fig. 1A and 2B) from the response of PBMC cultured with each of the three peptides or rhMOG (Δcpm). As is shown in Figure 5, left panels, positive responses against the peptides were not consistently detected. Only in monkey A5, we observed proliferation against MOG_34–56_ after day 140, but this was after immunization with MOG_34–56_ in IFA. These findings suggest that *in vivo* activated T-cells did not respond to peptides presented by regular APC present in PBMC.

When peptide-pulsed autologous B-LCL were added to the cultures, a clear specific response against the presented peptide could be detected, which was superimposed on the background response of PBMC against unpulsed B-LCL. The background response ranged between 2,700 and 106,000 cpm, but was highly variable between monkeys and over time. Positive responses against the peptides used for pulsing the B-LCL were detected in each of the groups in several animals (Fig. 5, right panels), generally first appearing around day 70, which was well before the booster immunization. Note that the peptide-specific proliferative responses in groups A and B were not further enhanced by the booster immunization. This again indicates that the *in vivo* activated T-cells may preferentially respond to peptide presented on B-LCL.

### Autoantibody Responses

Sera were collected every two weeks starting prior to the B-LCL infusion on day 0. All sera were tested for reactivity with ELISA-plate bound MOG_34–56_, CMVmcp_981–1003_, cMOG_34–56_, and rhMOG. The criterion for a positive antibody response is a 1.5 times higher light absorbance at two or more consecutive time points after the B-LCL infusion compared to the day zero sample. Clear antibody reactivity was found in the monkeys from groups A and B, but only after the booster immunization with.

MOG_34–56_/IFA (Table 2). In three animals weak antibody binding was detected before the booster immunization: monkey B3 transiently displayed a positive IgG response against CMVmcp_981–1003_ and rhMOG, monkey C1 had a positive response against rhMOG and monkey C2 developed positive IgG responses as early as day 14 against all four antigens tested. After the booster immunization IgG antibodies against MOG_34–56_ and rhMOG could be detected in two monkeys from group A and 4 from group B. IgG binding to citrullinated MOG_34–56_ was detected, but at a lower level than to MOG_34–56_.

**Table 2:** IgG responses.

Group	Animal	MOG_34–56_	CMVmcp_981–1003_	cMOG_34–56_	rhMOG
A (MOG_34–56_)	A1	−	−	−	−
	A2	++	−	−	**++**
	A3	−	−	−	−
	A4	−	−	−	−
	A5	+++	−	**+**	**+++**
B (CMVmcp_981–1003_)	B1	++	−	**-**	**+**
	B2	+	−	−	**+**
	B3	−	+*	−	+*
	B4	++	−	+	++
	B5	++	−	+	++
C (cMOG_34–56_)	C1	−	−	−	+*
	C2	+*	+*	**+** *	**+** *
	C3	−	−	−	−
	C4	−	−	−	−
	C5	−	−	−	−

* Positive responses developed before the booster immunization on day 98.

A positive antibody response was defined as the light absorbance at 405 nm being >1.5 times than the absorbance of the day-0 sample on at least two time points.

In conclusion, autoantibodies were not always detected and those present reacted only poorly with rhMOG protein. A prominent pathogenic contribution of these antibodies is therefore unlikely.

### CNS Pathology Induced by the Infusion of Citrullinated MOG Peptide-pulsed B-LCL

**Figure 6:**
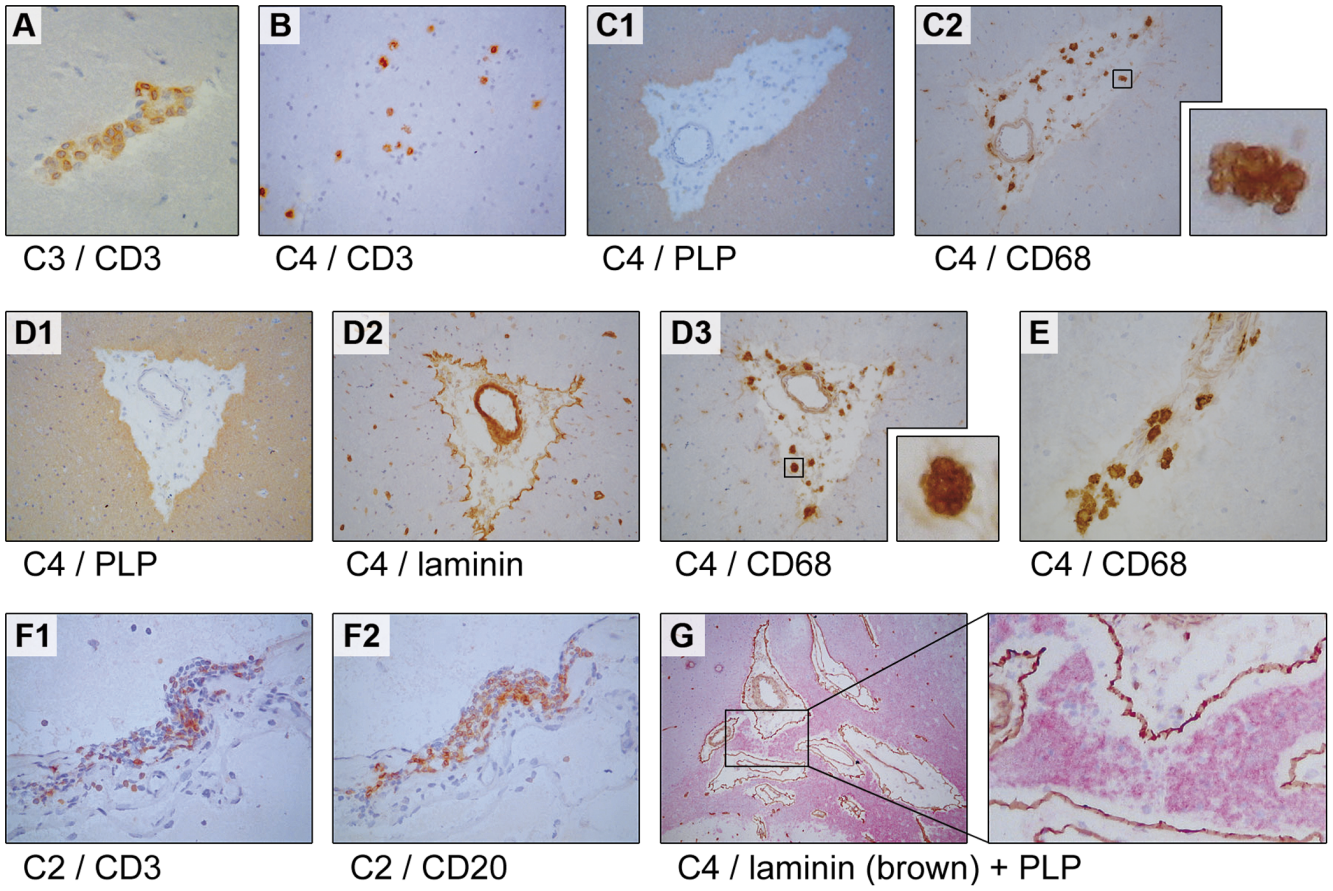
Histological features induced with cMOG_34–56_-pulsed B-LCL (group C). Indicated beneath each panel is the animal and the staining displayed in the respective panel. In all monkeys from group C and in some monkeys from groups A and B perivascular cuffs of infiltrated CD3^+^ T-cells could be found, an example of which is given in panel A. Diffuse infiltrates of CD3^+^ T-cells were more rarely found (B). Only in 3 monkeys lesions of relatively large size were found (C, D and E). (C) Shown is one lesion with PLP staining (C1) with infiltrated CD68^+^ macrophages (C2). The laminin staining in D2 shows that the macrophages remain confined to the Virchow Rubin space and do not pass the glia limitans. The inserts to C2 and D3 show the typical CD68 staining pattern we found. Infiltrates of CD3^+^ and CD20^+^ cells were also found in the meninges, where the two cell types seemed to co-localize (F1, F2). In monkey C4, in which the largest lesions were found, we observed some areas of myelin degeneration (G). It is unclear whether this represents the start of demyelination.

T-cells specific for MOG_34–56_ were identified as the core autoreactive mechanism for the induction of EAE in primates [17], [19]. The question is therefore warranted whether the *in vivo* activation of MOG_34–56_– reactive T-cells by infusion of peptide-pulsed B-LCL had elicited inflammation within the CNS white matter. We compared the brains from the three experimental groups with brains obtained from animals that had not been immunized with CNS antigens; some had participated as control animals in a collagen-induced arthritis study and had for this reason been immunized with bovine type II collagen in CFA. The results are summarized in Table 3 and representative histological pictures are shown in Figure 6.

**Table 3:** CNS histopathology.

Group	Perivascular CD3^+^ cell clusters	CD3^+^ cluster in meninges	CD3^+^CD20^+^ cluster in meninges	Perivascular edema with CD68^+^ cells
Normal brain	0/7	0/7	0/7	0/7
A (MOG_34–56_)	2/5	1/5	1/5	1/5
B (CMVmcp_981–1003_)	3/5	1/5	0/5	1/5
C (cMOG_34–56_)	5/5	4/5	2/5	2/5

Shown is the occurrence (number of animals/total number of animals) of perivascular CD3^+^ cell, CD3+ and CD3^+^CD20^+^ (in brackets) clusters in the meninges and perivascular edema with CD68^+^ cells. Positive animals were identified as having several clusters containing >4 positive cells in more than one location.

We observed a small cluster containing CD3^+^ T- and and CD20^+^ B-cells in the ventricle of one of the 5 animals of group A (monkey A3; not shown). In addition, small-sized perivascular cuffs, such as shown in Figure 6A, could be detected in the brain parenchyma of animals A3 and A5.

In 3 animals of group B (B2, B3, B4) we observed similar patterns of small-sized perivascular cuffs in the brain parenchyma (not shown) as observed in group A monkeys. Such histological signs of mild CNS inflammation were also previously found in monkeys sensitized against MOG_34–56_ in IFA [17]. Hence, these rather unremarkable pathological findings may be caused by the booster-immunization with MOG_34–56_ in IFA rather than by the infused B-cells. Although a few CD3^+^ cells can be found in normal brain in vessel wall (not shown), these were always less than 4 cells per vessel and were never found in a cluster-like organization as observed in group A or B animals.

The most remarkable changes were observed in group C monkeys, which had received B-LCL pulsed with citrullinated MOG_34–56_, but were not sensitized against MOG_34–56_/IFA; these were euthanized at ±42 days after the last B-cell transfer. In all animals perivascular clusters of CD3^+^ cells were found scattered throughout the parenchyma. In 4 of the 5 animals we found clusters of CD3^+^ T-cells in the meninges; two of which also contained CD20^+^ B-cells. In addition 3 of the 5 animals (monkeys C2, C3, C4) showed enlarged perivascular spaces as shown by laminin staining of the vessel wall and glia limitans.

Representative examples of the intrameningeal clusters of CD3^+^ and CD20^+^ cells are shown in Figure 6F. Occasionally co-localisation of CD3^+^ and CD20^+^ was found. In monkey C4 we observed relatively large areas of perivenular inflammation within the brain parenchyma, containing oedema and infiltrated CD68^+^ macrophages (Fig. 6C and D). Such large inflammatory lesions were occasionally associated with myelin degeneration (Fig. 6G). The CD68 staining was different from the typically homogenous cytoplasmic staining of macrophages, as we observed a patchy staining pattern (Fig. 6D and E), suggesting that the antibody may be bound to phagocytosed material. CD68 is a receptor of lipoprotein often found co-localized with lysosome-associate membrane proteins in lysosomal/endosomal compartments [28]. This patchy staining pattern is also reminiscent of the foamy macrophages in MS brains and those generated *in vitro* by feeding macrophages myelin [29].

The infiltration of tissues with B-LCL may be a non-specific phenomenon, raising the possibility that B-LCL may also be found in other tissues. We investigated whether mononuclear cell infiltrates could be in the livers of monkeys from group C, which had the most prominent infiltrates in the brain (monkeys C2, C3, C4). Indeed, we detected infiltrated CD3 and CD20+ cells in the liver in monkey C2 (Fig. S1), but not in the other two monkeys.

## Discussion

The main message of this publication is that intravenous injection of autologous B-LCL (3 doses at 28 days intervals) pre-pulsed with the modified self-peptide (citrullinated) MOG_34–56_ elicits activation of T-cells present in the naïve repertoire. The *in vivo* activated T-cells cross-react *ex vivo* with the parent peptide MOG_34–56_ and the viral peptide CMVmcp_981–1003_, which shares a mimicry motif with MOG_34–56_. These T-cells, which had been activated *in vivo* by peptide-pulsed B-LCL, could not be reactivated *ex vivo* with peptide presented by circulating APC. However, when the peptide was presented by B-LCL, reactivation was observed.

The second message is that monkeys sacrificed 6 weeks after the final infusion of citrullinated MOG_34–56_ pulsed B-LCL, displayed pathological signs of neuro-inflammation. Different from the common appearance of lesions observed in EAE models, but more comparable with those observed in MS patients, these lesions are populated with moderate numbers of inflammatory cells. The prevalent cells in these lesions were macrophages characterized by patchy staining with CD68, a staining pattern also observed in foamy macrophages. In the meninges of several monkeys we detected clusters of CD3^+^ T-cells and CD20^+^ B-cells, although it is unclear at this stage whether the CD20^+^ B-cells are LCV infected.

A technical problem has been that the numbers of B-LCL available for infusion varied considerably between animals. This is inherent to the outbred nature of the rhesus monkey model. Individuals are MHC incompatible, thus only autologous APC can be used for T cell stimulation. Poor growth of B-LCL lines from some monkeys is likely due to infection with simian foamy virus (SFV). SFV infects rhesus monkeys without causing clinical symptoms, but impairs growth of cell lines. This is a new model, so we had no *a priori* indication which B-LCL numbers were needed to induce a response. We detected no correlation between the numbers of infused B-LCL with any of the outcome parameters, thus we believe that the variation in infused B-LCL numbers did not have a major influence on the outcome of the study.

The B-LCL themselves do not contain a preference for the brain; they may infiltrate other tissues as well. We investigated the livers of the three monkeys of group C and found infiltrates in one of them (C2, Figure S1). Supposedly, T-cells are attracted to the site where B-LCL have infiltrated. Only if they have the right specificity, T-cells can cause tissue destruction. In our study, we have elicited MOG peptide-specific T-cell responses, so these exert their pathogenic responses in the brain.

In group A and B monkeys, which were sacrificed substantially later, to examine the effect of additional immunization with MOG_34–56_/IFA, such conspicuous structures were not detected in the meninges, but instead we found small-sized perivascular infiltrates. This implies that immunization with MOG_34–56_ in IFA most likely does not support the pathogenic function of the T-cells induced with peptide-pulsed B-LCL.

The neuropathological alterations observed in the monkeys infused with citrullinated MOG_34–56_ pulsed B-LCL are normally only found in the CNS of monkeys affected by EAE, indicating that the activated T-cells may have comparable encephalitogenic capacity to T-cells activated in the EAE model induced with MOG_34–56_ in adjuvant [17]. The *in vivo* activated T-cells were further examined *ex vivo*, demonstrating that the induced peptide-specific T-cell reactivity was superimposed on a variable background proliferation by CD3^+^CD8^+^ (CTL), CD3^+^CD8^+^CD56^+^ (NK-CTL) and CD3^−^CD56^+^ (NK cells) against the B-LCL. T-cells cross-reactive with the baboon LCV infected B-LCL were already present in the pre-immune repertoire of the monkeys (Figure 1). We assume therefore that the background proliferation is due to T-cells engaged in controlling the natural infection with the monkey’s own macaque LCV.

To investigate whether the observed effects were indeed caused by the fact that the B-cells were transformed, the control experiment would be to infuse untransformed B-cells. However, because each monkey in our colony is genetically unique, B-cells would have had to be isolated from each individual recipient. Several months would be needed to obtain numbers of B-cells equivalent to the number of B-LCL used in the study. These autologous B-cells would have had to be isolated by cell sorting and would have had to be stored frozen before the start of the study. Altogether, we considered that the many technical hurdles would reduce the chance that meaningful results could be obtained; hence we chose not to include this control.

Peptide-specific antibody responses were not consistently detected before the boost. However, antibody responses against MOG_34–56_ and rhMOG were boosted in groups A and B by the immunization with MOG_34–56_ in IFA on day 98. This could be a primary response to the booster, but in a previous study [17] only one out of three animals immunized with CMVmcp_981–1003_ in CFA and boosted with MOG_34–56_ in IFA (the latter identical as in our study) developed low amounts of antibodies to rhMOG, and none of the three animals developed antibodies against MOG_34–56_, whereas they all developed antibodies against CMVmcp_981–1003_ (unpublished results from the study by Brok et al. [17]). In our study 5 out of 5 monkeys infused with CMVmcp_981–1003_ pulsed autologous B-LCL followed by immunization with MOG_34–56_ in IFA developed antibodies against rhMOG and 4 out of 5 against MOG_34–56_. Note that one animal had already developed antibodies against rhMOG before the immunization. Possibly, the presentation of CMVmcp_981–1003_ by B-LCL, in contrast to immunization with CMVmcp_981–1003_ in CFA primes anti-MOG antibody production, which is subsequently boosted after the MOG_34–56_ in IFA immunization. Given the molecular mimicry motif shared between CMVmcp_981–1003_ and MOG_34–56_ this is not an unlikely explanation.

In the past years we have obtained several lines of evidence, mainly from the EAE model in marmosets, that MOG_34–56_ reactive T-cells represent an uncommon type of T-cells. First, immunization with MOG_34–56_ in IFA is a sufficient stimulus for their *in vivo* activation and the exposure of their remarkable encephalitogenic potential [19]. Second, the specific T-cell epitope in MOG_34–56_ has been defined at MOG_40–48_, representing a mimicry motif shared with the UL86 ORF encoded major capsid protein of human and rhesus monkey cytomegalovirus [17]. This finding led us to hypothesize a relation between the autoreactive T-cells that cause clinically evident EAE and anti-CMV memory T-cells [30]. Also the phenotype of MOG_34–56_ induced T-cell lines (CD3^+^CD4/8^+^CD56^+^CD16^−^CD45RO^-^CD27^+^CD28^−^) [31], points to CMV, as it is reminiscent to the natural killer-like cytolytic T-cell subset engaged in the control of CMV latency [32], [33]. Taken together, these features led us to hypothesize that the MOG_34–56_ reactive T-cells that mediate the CNS targeting autoimmune attack in non-human primate EAE models may be related to an elusive CMV-specific subset of “resting vigilant T-cells” [34].

We chose not to include a group infused with autologous B-LCL not pulsed with antigen, as such a group would not enable measuring the induced peptide specific response and thus the effect of the infusion. However, the results reported in this publication show that our model system worked and the experiment was repeated in five marmoset twins [35]. One sibling received autologous B-LCL pulsed with MOG_34–56_ and the other sibling received non-pulsed autologous B-LCL. PBMC from the siblings in the B-LCL/MOG_34–56_ group produced more IL17A when stimulated with MOG than PBMC from siblings in the non-pulsed B-LCL group. In two monkeys of the B-LCL/MOG_34–56_ group infiltrates of CD3^+^ and CD20^+^ cells were observed in the meninges, whereas these were not found in their fraternal siblings. These results further indicate that MOG peptide pulsed B-LCL induce T-cell responses in the brain.

The fact that we have not observed marked CNS injury and overt neurological deficit in this three-month experiment may be a factor of time. While in actively induced EAE models development of lesions and neurological deficit can be observed within a period of a few weeks to months after initiation, this is clearly not the case in MS. This implies that considerably longer observation periods than the three months of the current study may have to be maintained to enable complete unfolding of the clinical and neuropathological symptoms.

In conclusion, this publication presents a possible mechanistic explanation for the relation between EBV infection and MS. We propose that by the infection with LCV, B-cells acquire the capacity to recruit a specific population of MOG_34–56_ specific T-cells from the immune repertoire. T-cells of this specificity were found to mediate CNS inflammation in both the rhesus monkey and the marmoset EAE models. It is tempting to speculate that the unravelling of mechanisms employed by the B-LCL for activation of the encephalitogenic T-cells may provide new targets of therapy in MS.

## Supporting Information

Figure S1 **Perivascular infiltrates found in the liver of monkey C2.** (A, E) HE staining showing two areas of infiltrated cells in the liver. (B, D) magnification of infiltrated areas (200 and 400×, respectively). These areas have been stained with CD3 (C, G) and CD20 (D, H), in subsequent slides, also magnified 200 and 400×, respectively.
(DOCX)
