# Supplementary material for: Induction of Encephalitis in Rhesus Monkeys Infused with Lymphocryptovirus-Infected B-Cells Presenting MOG34–56 Peptide
Source: PLoS One. 2013 Aug 15;8(8):e71549. doi: 10.1371/journal.pone.0071549 (PMC3744571; doi:10.1371/journal.pone.0071549)
Supplement: Figure S1 — Perivascular infiltrates found in the liver of monkey C2. (A, E) HE staining showing two areas of infiltrated cells in the liver. (B, D) magnification of infiltrated areas (200 and 400×, respectively). These areas have been stained with CD3 (C, G) and CD20 (D, H), in subsequent slides, also magnified 200 and 400×, respectively. (DOCX) [file pone.0071549.s001.docx]

Supplemental Figures


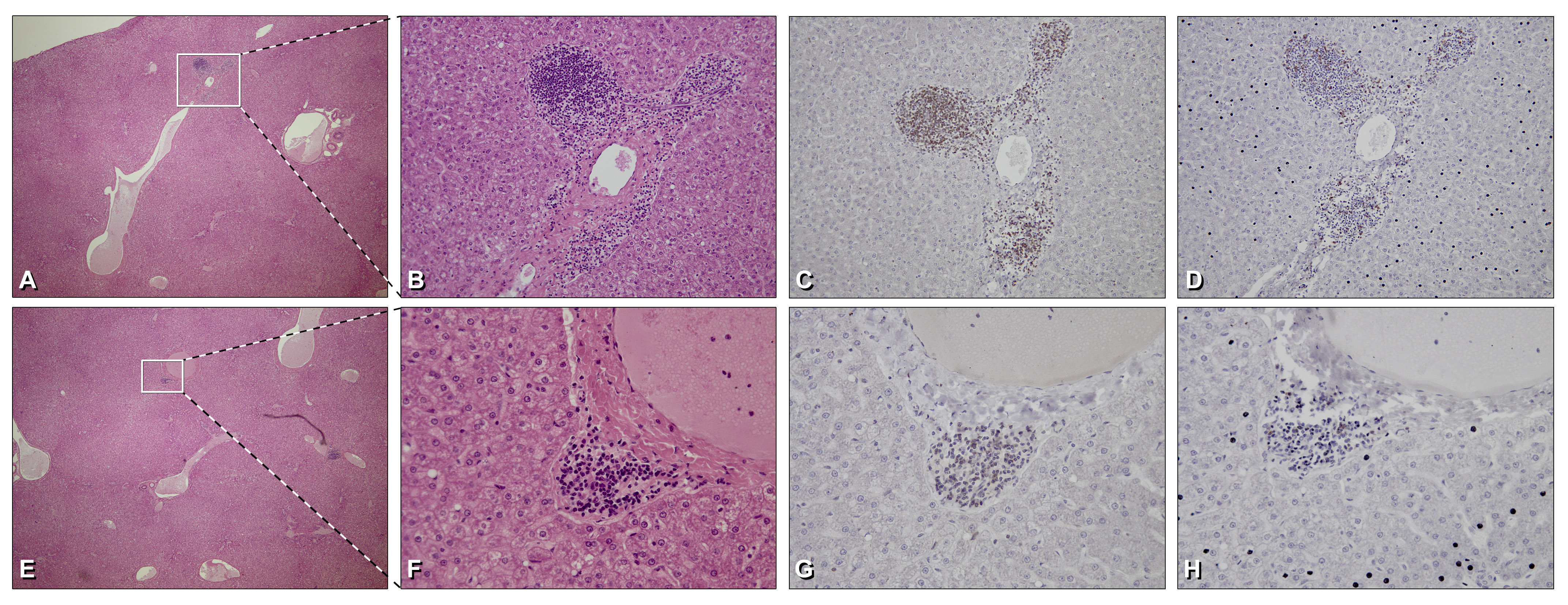


**Figure S1. Perivascular infiltrates found in the liver of monkey C2.** (A, E) HE staining showing two areas of infiltrated cells in the liver. (B, D) magnification of infiltrated areas (200 and 400x, respectively). These areas have been stained with CD3 (C, G) and CD20 (D, H), in subsequent slides, also magnified 200 and 400x, respectively.
